# Recent Patents of Interest to Dentists

**Published:** 1907-07

**Authors:** 


### PATENTS.

### RECENT PATENTS OF INTEREST TO DENTISTS.

854283. Dental root impression and crown mounting instrument, John M. Evey, Monmouth, Ill.

854486. Display carrier for artificial, Joseph F. Gibson, York, Pa.

853984. Tooth crown model, Chapin F. Lauderdale, Milwaukee, Wis.

854116. Toothpick machine, George P. Stanley and W. W. Tainter, Dixfield, Maine.

855288. Hand piece for dental engines, Frederick W. Dean, Des Moines, Iowa.

854898. Mouth brace, Hans Lorenz, Rochester, N. Y.

854842. Artificial tooth, Joseph Ramsperger, York, Pa.

855875. Dentimeter, Joseph Bode, Philadelphia, Pa.

856034. Manufacturing dental fillings, inlays and crowns, John N. Crouse, Chicago, Ill.

856884. Attachment for dental articulators, Milton H. Knapp, Saginaw, Mich.

38620. Design, side frame for dentist and barber chairs, James Barker, Philadelphia, Pa.

857339. Dental plate swage, Grant E. Freeborn, Belfast, N. Y.

857240. Dental tray, James A. Henning, Beardstown, Ill.

857414. Tooth powder box, Walter H. Perkins, Waterbury, Conn.

Copies of above patents may be obtained for fifteen cents each by addressing John A. Saul, Solicitor of Patents, Fendall Building, Washington, D. C.

